# Clarifying sub-genomic positions of QTLs for flowering habit and fruit quality in U.S. strawberry (*Fragaria*×*ananassa*) breeding populations using pedigree-based QTL analysis

**DOI:** 10.1038/hortres.2017.62

**Published:** 2017-11-08

**Authors:** Sujeet Verma, Jason D Zurn, Natalia Salinas, Megan M Mathey, Beatrice Denoyes, James F Hancock, Chad E Finn, Nahla V Bassil, Vance M Whitaker

**Affiliations:** 1 IFAS/Department of Horticulture, University of Florida, Gulf Coast Research and Education Center, 14625 CR 672, Wimauma, FL 33598, USA; 2 USDA-ARS National Clonal Germplasm Repository, 33447 Peoria Road, Corvallis, OR 97333, USA; 3 Department of Horticulture, Oregon State University, 4017 Agriculture and Life Sciences Building, Corvallis, OR 97331, USA; 4 Department of Horticulture, UMR 1332 Biologie du Fruit et Pathologie, INRA, Univ. Bordeaux, Villenave d’Ornon F-33140, France; 5 Michigan State University, East Lansing, MI 48824, USA; 6 USDA-ARS, HCRU, 3420 NW Orchard Avenue, Corvallis, OR 97330, USA

## Abstract

The cultivated strawberry (*Fragaria*×*ananassa*) is consumed worldwide for its flavor and nutritional benefits. Genetic analysis of commercially important traits in strawberry are important for the development of breeding methods and tools for this species. Although several quantitative trait loci (QTL) have been previously detected for fruit quality and flowering traits using low-density genetic maps, clarity on the sub-genomic locations of these QTLs was missing. Recent discoveries in allo-octoploid strawberry genomics led to the development of the IStraw90 single-nucleotide polymorphism (SNP) array, enabling high-density genetic maps and finer resolution QTL analysis. In this study, breeder-specified traits were evaluated in the Eastern (Michigan) and Western (Oregon) United States for a common set of breeding populations during 2 years. Several QTLs were validated for soluble solids content (SSC), fruit weight (FWT), pH and titratable acidity (TA) using a pedigree-based QTL analysis approach. For fruit quality, a QTL for SSC on linkage group (LG) 6A, a QTL for FWT on LG 2BII, a QTL for pH on LG 4CII and two QTLs for TA on LGs 2A and 5B were detected. In addition, a large-effect QTL for flowering was detected at the distal end of LG 4A, coinciding with the *FaPFRU* locus. Marker haplotype analysis in the *FaPFRU* region indicated that the homozygous recessive genotype was highly predictive of seasonal flowering. SNP probes in the *FaPFRU* region may help facilitate marker-assisted selection for this trait.

## Introduction

The cultivated strawberry (*Fragaria*×*ananassa* Duch. ex Rozier) is widely consumed fresh or in processed form. Improved fruit quality is desired by consumers and sought by breeders.^1^ Major components of fruit quality attributes include appearance, texture, taste, aroma and nutritional content.^2^ The intense flavor of strawberry is characterized by high titratable acidity (TA), high soluble solids content (SSC) and strong aroma.^3^ Some progress toward selecting for aroma compounds and flavor has been made over the last few years.^3,4^ In addition to good flavor, strawberries contain antioxidant and other nutritional compounds that may help reduce risk of some chronic ailments,^5–7^ and there has been substantial research aimed at identifying the genes involved in the biosynthetic pathways involved in the accumulation of these compounds.^8–10^

Cultivated strawberries are allo-octoploid (2*n*=8×=56) and mapping studies show major disomic inheritance.^1,11^ In the last decade, numerous quantitative trait loci (QTLs) for strawberry fruit quality traits have been detected across diverse breeding material. Lerceteau-Köhler *et al.*^12^ pioneered QTL mapping in octoploid strawberry, detecting several QTLs for 19 fruit development and quality traits in a cross between ‘Capitola’ and the selection CF1116. Most of the QTLs explained 10–17% of the total phenotypic variation for the corresponding trait. Low genetic effects for fruit quality traits are expected as they are strongly influenced by environmental conditions.^13^ Zorrilla-Fontanesi *et al.*^3,14^ detected several QTLs for fruit quality traits and volatile compounds in a cross between the Spanish selections 232 and 1392. Castro and Lewers^15^ used 177 F_1_ individuals from a cross between ‘Delmarvel’ and ‘Selva’ to conduct QTL analysis for fruit quality traits and identified QTLs in many of the same regions identified by Lerceteau-Köhler *et al*.^12^

Flowering behavior is another important trait in strawberry plants and is strongly affected by temperature and photoperiod conditions.^16–18^ Strawberry plants are classified into seasonal flowering (SF also known as ‘short-day’ or ‘June-bearing’) or perpetual flowering (PF also known as ‘everbearing’, ‘day-neutral’ or ‘remontant’). SF plants typically initiate flower buds under photoperiods of <14 h or at temperatures below 15 °C and usually have a shortened harvest period compared with PF plants.^15,17^ Conversely, PF strawberries can initiate flowering when temperatures are <30 °C during the day all summer and into the autumn.^19,20^ An extended harvest season is an attractive quality for a strawberry, particularly in the central coast of California and other summer production regions. Numerous sources of the PF trait have been identified in octoploid strawberry.^19,20,21^ However, only the PF phenotype conferred by the *FaPFRU* locus from a *F. virginiana* subsp. *glauca* accession originating from the Wasatch Mountains in Utah has been studied in great detail.^15,16,18,22–27^ This single dominant locus confers the ability to initiate flowers in *F.* ×*ananassa* under long days at permissive temperatures and is located on the *F. vesca* chromosome 4 homolog.^15,16,23,25^ Recently, Honjo *et al.*^28^ conducted QTL analysis in three populations segregating for PF that were derived from three different PF cultivars. Two of the PF cultivars, ‘Summer-berry’ and ‘Ever-berry’, are cultivars classified as ‘older everbearers’ and are derived from an unknown Japanese PF source while ‘Hecker’ is a day-neutral cultivar developed at the UC Davis program with the *FaPFRU* locus.^21,28^ A QTL located on chromosome 4 was detected in all three PF cultivars and a subsequent allelism test suggested the PF phenotype in the Japanese cultivars is controlled by a tightly linked gene or the same gene as that present at *FaPFRU*.^28^ Salinas *et al.*^18^ identified a diagnostic allele for the *FaPFRU* locus in the PF Japanese cultivar Ooishi-shikinari 2, which is derived from the same source as ‘Ever-berry’, and suggested that the *FaPFRU* locus may have been introgressed numerous times before its initial discovery by Bringhurst in 1955.

There were ambiguities in clear sub-genome assignments of QTLs in the past and this study uses advanced molecular tools to refine and clarify those ambiguities. In each of the previous QTL studies, low-density genetic maps in bi-parental populations were used for QTL detection and sub-genome identification. These low-density maps often contained different marker types (amplified fragment length polymorphism and simple sequence repeats (SSRs)) and as a result, the studies are difficult to compare. In addition to that, in the past, QTLs were mostly assigned to a homeologous group (HG) rather than a specific sub-genome. Moreover, bi-parental populations often exhibit limited genetic diversity, resulting in only a few alleles within the germplasm being identified.^29,30^ To maximize gain of genetic information, a QTL analysis approach using multiparental pedigree-linked strawberry populations was developed^29,30^ and in conjunction with the Axiom IStraw90 SNP array^31^ has become a powerful tool for high-resolution QTL mapping.^32,33^ Using this pedigree-based QTL analysis (PBA) approach, implemented through FlexQTL, provides advantages over traditional bi-parental population methods, allowing for a QTL to be evaluated in numerous genetic backgrounds while simultaneously increasing the chances for recombination events near QTLs controlling traits of interest.^29,30^ The IStraw90 array^31^ allows for the high-throughput, repeatable genotyping necessary to produce a high-quality consensus map and clear sub-genome assignments. The genetic map used in this study follows sub-genome assignments from Koehorst-vanc Putten and van de Weg (2017, unpublished data), which clearly defines and separates octoploid strawberry sub-genomes. Individually, the bi-allelic single-nucleotide polymorphism (SNP) markers on the array provide limited information on the segregation of marker alleles via pedigrees because of potential homozygosity at these loci. However, when the markers are analyzed jointly, haplotypes useful for marker-assisted selection (MAS) can be identified.^34^

PBA is especially attractive in clonally propagated plants as statistical power can be increased by phenotypically evaluating ancestral germplasm in the same environments as descendants.^30^ When using PBA, it is critical to use a dense map as low marker density can add ambiguity in the transfer of alleles through generations.^29^ FlexQTL implements a Bayesian statistical approach using pedigree-linked populations to improve statistical power and detect vertically inherited QTL alleles.^29^ Confirmation of accurate pedigree information is particularly important when utilizing PBA, and pedigree records from a breeding program must be checked for accuracy because of human error or pollen contamination.^29,30^ FlexQTL traces founder alleles to contemporary generation and if pedigree records entered incorrectly, it provides information on which pedigree records are associated with inconsistencies.

The objectives of this study were to further refine and clarify the sub-genomic locations of major fruit quality and flowering habit QTLs, to dissect the genetics controlling fruit quality and flowering in *F.* ×*ananassa* using the Axiom IStraw90 SNP array for genotyping together with FlexQTL for QTL analysis of multiparental pedigree-linked populations, and to compare the QTL results with previous studies. SNP haplotype analysis was applied to define the locus of a trait controlled by one strong and consistent QTL for PF detected by FlexQTL.

## Materials and methods

### Breeding germplasm

This study focused on breeding populations established in the RosBREED Project to represent the Michigan State University (MSU) and the USDA-ARS Horticultural Crops Research Unit (HCRU-indicated by Oregon U.S. (ORUS)) strawberry breeding programs in Michigan (MI) and Oregon (OR), respectively.^35^ The pedigree-connected germplasm consisted of 23 F_1_ families representing 11 important breeding parents from the two breeding programs and their 249 progeny, collectively (Supplementary Information S1). The 11 parents included the PF parents ‘Tribute’, ‘Fort Laramie’ and ‘Seascape’ and the SF ‘Earliglow’, ‘Honeoye’, ‘Puget Reliance’, ‘Totem’ and selections, MSU 49 and MSU 56.

### Phenotypic data

Phenotypic data for seven fruit quality traits were measured or scored in 2011 and 2012 in MI and OR according to Mathey *et al.*^35^ and included: SSC (°Brix), fruit weight (FWT) (g per fruit), firmness (Firm; 1 (very soft) −9 (very firm) scale), TA (g L^–1^ citric acid), ratio of SSC and TA (SSC:TA), pH, flavor and external color (ExtCol). In addition to the fruit quality traits, the ability of an individual to perpetually flower (PF) was assessed as described by Mathey *et al*.^35^ In this study, flowering was recorded as a binary trait, 0 for SF and 1 for PF, based on the presence of flowers on or after 17 July.^35^ Phenotyping at two locations and over 2 years allowed testing of genetic control of traits in different environments and years. Density plots were visualized for each trait. Spearman’s rank correlation coefficients (r) and *P* values were adjusted for multiple comparisons using the Bonferroni correction method for each trait over 2 years using R version 3.2.5.^36^

### Genotyping and genetic map

DNA from seedlings and parents was extracted from actively growing leaf tissue with the E-Z 96 Plant DNA extraction kit (Omega BioTek, Norcross, GA, USA) as previously described.^37^ The resulting genomic DNA was quantitated with the Quant-iT Picogreen Assay (Invitrogen, Eugene, OR, USA) according to the manufacturer’s recommendations using a Victor^3^V 1420 Multilabel Counter (Perkin Elmer, Downers Grove, IL, USA). DNA concentrations were adjusted to 20–50 ng μL^–1^ and submitted to Affymetrix, Inc. (Santa Clara, CA, USA) for genotyping with the Axiom IStraw90 SNP array.^31^ Genotypic data acquired from Affymetrix, Inc. was recoded with GenomeStudioConverter^34^ to convert it into the software input file format needed for Pedimap version 1.2^38^ and FlexQTL version 099130.^29^ Genetic positions of 10 560 filtered *PolyHighResolution* and *NoMinorHomozygous* SNP markers identified in Bassil and Davis *et al.*^31^ were obtained from a high-density SNP genetic map constructed using the ‘Holiday’ × ‘Korona’ population (Koehorst-vanc Putten and van de Weg, 2017, unpublished data). Sub-genome assignments and orientation of each linkage group (LG) was based on van Dijk *et al*.^39^ The homozygous regions across parents were mapped using R software to identify genetic regions where lower QTL detection power would be expected.^36^

### Pedigree confirmation

Genotypic data were tested for pedigree relationships and marker inheritance errors using phased marker allele information from FlexQTL version 099130.^29^ Extensive checking of parentage records and marker calls were conducted using SNP marker data and a Microsoft Excel-based tool (van de Weg E, 2016, unpublished data). The Excel-based tool was utilized to identify and eliminate off-type individuals and to identify correct parentage. In general, marker genotypes were filtered for consistency of calls and incorrect pedigree records were corrected after validation. When genotypic data were not available for the parents, they were predicted with FlexQTL and the PediHaplotyper R package,^38^ and then used to confirm pedigree relationships.

### QTL detection and haplotyping

Haploblock (HB) alleles^29,38^ were created using the R package *fp_haplotyper* (Roeland Voorrips, 2014, unpublished data) and 10 560 SNP markers, in order to save computation time. FlexQTL was used for genome-wide QTL analysis using HB alleles.^29,34^ LGs where significant QTLs were identified in at least two out of four location-years were reanalyzed with SNP markers using FlexQTL.^32,40–43^ Phenotypic data of only unselected material (seedlings) were used for QTL analysis to prevent prediction biases. Parameter settings used for all FlexQTL runs followed Mangandi *et al*.^33^ The Bayes factor parameter (2lnBF) was interpreted as nonsignificant (0–2), positive (2–5), strong (5–10) or decisive (>10) evidence for the presence of QTLs. Genome-wide evidence was collected in support of QTL models ranging from 1 QTL versus 0 QTLs to *n* versus *n*-1 QTLs, where *n* is the number of QTLs detected in the model. MapChart version 2.3^44^ and VisualFlexQTL version 0.1.0.42 were used to visualize FlexQTL output on traces of convergence of QTL models to identify the genomic regions associated with significant QTLs. Additional posterior inference analysis, using the postQTL module, was conducted to delineate significant and repeatable QTL regions in order to obtain the best posterior estimates of genotype probabilities, breeding values and weighted additive variances. The phenotypic variation explained by a QTL was estimated as PVE (%)=[(probability×AVt)/Vp]×100, where probability is the cumulative probability of presence of a QTL in the interval, *AVt* is the additive variance for the trait, and *V*_P_ is the total phenotypic variance. QTL effects were calculated and reported.

### Assessment of EMFv006 marker for DNA informed breeding

The SSR marker EMFv006 was previously found to be associated with an SSC QTL on chromosome 6A.^12^ EMFv006 was assessed for potential use in MAS by genotyping these populations for which phenotypic data was also gathered. PCR was conducted using the Type-It Multiplex Microsatellite PCR Qiagen kit (Qiagen N.V., Venlo, The Netherlands) in a total volume of 15 μL. Each reaction consisted of 8.3 μL of 2X Type-it Multiplex PCR Master Mix, a fluorescently labeled forward primer and reverse primer each at a final concentration of 0.025 μM, 1.7 μL of Q-solution and 3.3 μL of 3 ng μL^–1^ DNA. Amplification was performed in a PTC-225 Thermal Cycler (MJ Research, Inc., Waltham, MA, U.S.A.) using a touch-down program that consisted of an initial denaturation of 95 °C for 5 min; 10 cycles of 95 °C for 30 s; 62 °C for 1.5 min decreasing by 1 °C per cycle; and 72 °C for 30 s; followed by 29 cycles of 95 °C for 30 s; 52 °C for 1.5 min; 72 °C for 30 s; and a final extension at 60 °C for 30 min. The PCR products were kept at 4 °C until removed from the thermocycler. PCR success was assessed by 2% agarose gel electrophoresis. Allele composition for this SSR marker was determined for each individual after separation of PCR products by capillary electrophoresis with a Beckman CEQ 8000 (Beckman Coulter, Inc, Pasadena, CA, USA). Beckman CEQ 8000 genetic analysis system software v 8.0.52 was used for allele visualization and scoring.

### Assessment of HB markers for MAS

SNP markers covering the QTL confidence interval region were chosen for haplotyping. Phased haplotype information was obtained for each individual from the FlexQTL output and the number of individuals represented by haplotypes and diplotypes by each of the 15 SNP markers was calculated using Excel. Haploview version 4.2^45^ was used to identify recombination hotspots and HBs within the significant QTL regions that were detected in multiple location-years. PediHaplotyper R package^34^ was used to haplotype significant QTL regions. Flanking sequences of the SNP markers in the QTL region of *FaPFRU* were BLAST searched against the NCBI nucleotide database and the scaffolds of the *F. vesca* v1.0 genome assembly^46^ to confirm marker groupings. SNP markers and SSRs associated with *FaPFRU* were physically mapped to the identified *F. vesca* scaffolds using Bowtie2 version 2.2.9^47^ and SAMtools version 1.3.1,^48^ and visualized using the Integrative Genomics Viewer version 2.3.82.^49,50^ Contingency tables were constructed for each of the haplotypes using different diagnostic interpretations of the genotypic data. The summary statistics of accuracy, the positive predictive value, the negative predictive value, sensitivity, specificity and the adjusted diagnostic odds ratio were calculated for each test interpretation and the best interpretation for MAS was identified.^18,51,52^

## Results

### Phenotypic data

**Figure 1:**
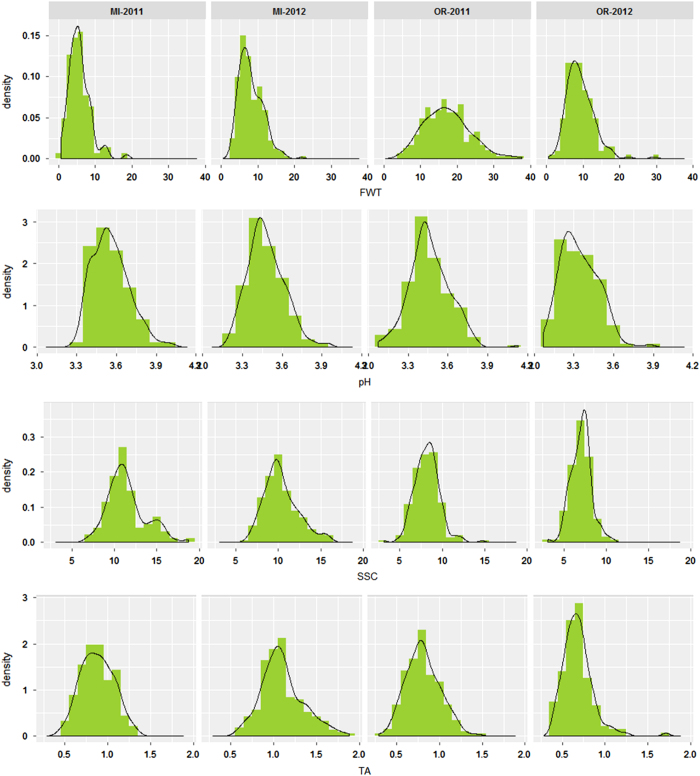
Density plots of FWT, pH, SSC and TA for MI and OR in 2011 and 2012 indicate that many of the trait measurements exhibited an approximately normal distribution density fit lines as indicated in black.

The phenotypic distribution of many of the fruit quality traits approached normal distributions (Figure 1, Supplementary Figure S1). Mean FWT observed in OR in 2011 and 2012 (16.9 and 9.4 g) was higher than mean FWT observed in MI in 2011 and 2012 (5.4 and 7.8 g; Supplementary Table S1). The lowest FWT was observed in MI in 2011 (0.5 g) and the highest FWT was observed in OR in 2011 (37.6 g). Higher mean SSC was observed in MI in 2011 and 2012 (11.4 and 10.3 °Brix) compared with the mean SSC observed in OR in 2011 and 2012 (8.3 and 7.1 °Brix). Higher mean TA was observed in MI in 2011 and 2012 (0.9 and 1.1 g L^–1^ citric acid) compared with the mean TA observed in OR in 2011 and 2012 (0.81 and 0.69 g L^–1^ citric acid). Higher mean pH was observed in MI in 2011 and 2012 (3.5 and 4.5) compared with the mean pH observed in OR in 2011 and 2012 (3.4 and 3.3; Supplementary Table S1). Significant Spearman’s rank correlation coefficients (α=0.05) were observed for PF within a location across years and between locations within 2011 and 2012. Most of the correlations were not significant (α=0.05), however, significant negative correlation coefficients (r) were observed between TA and pH and significant positive correlations were observed for TA between locations (Supplementary Fig. S2).

### Pedigree confirmation and correction

Of the 23 MSU and ORUS families included in this study, genotypic data of most offspring matched the reported parents in 14 families, matched one of the parents in nine families (MSU 9–18, ORUS 3279, ORUS 3305, ORUS 3315, ORUS 3316, ORUS 3320, ORUS 3324, ORUS 3325 and ORUS 3326), and did not match either parent in one family (ORUS 3324) when using the Excel-based tool (Supplementary Information S1). Two genotypes of ‘Fort Laramie’ were predicted based on offspring genotypes where ‘Fort Laramie’ is the parent of MSU 9–8, MSU 9–9, MSU 9–10 and MSU 9–11 and ‘Fort Laramie 2’ is the parent of ORUS 3315, ORUS 3316 and ORUS 3324. An unknown pollen parent of MSU 9–18, which was supposed to be ‘Fort Laramie’, was also identified and is being referred to as father (F) of MSU 9–18. Two genotypes of ‘Puget Reliance’ were also predicted based on offspring genotypes where ‘Puget Reliance’ was the parent of ORUS 3315 and ORUS 3323, and ‘Puget Reliance 2’ was the parent of ORUS 3326. The SNP genotype of ‘Tillamook’ included in this study did not match that of offspring ORUS 3305, ORUS 3324 or ORUS 3325. The SNP genotypes of ‘Sarian’ and ORUS 2427–1 did not match that of their offspring in one population for each parent, ORUS 3320 for ‘Sarian’ and ORUS 3279 for ORUS 2427–1. After correcting the pedigree records of the 23 families, seven offspring were identified that did not match either parent’s genotype. These seven offspring were removed from further analysis (Supplementary Information S1).

### QTL analysis

A total of 28 LGs were constructed with varying number of sub-genomes (Supplementary Table S2). The length of the whole genome was 19.1 Morgans and LG 6C was the longest of all the groups extending to 128.7 cM. LG 5BII had the lowest number of markers, 14, and LG 6A had the largest number of markers, 677. The largest gap in the genetic map was of 24.5 cM on LG 7A (Supplementary Table S2). Polymorphic markers were well distributed among LGs and minor allele frequency ranged from 0 to 0.5 (Supplementary Figure S3).

QTLs for only four (SSC, FWT, pH and TA) out of the eight fruit quality traits were significant (Table 1; Supplementary Figures S4-S7). No QTLs were identified for fruit firmness (FIRM), ratio of SSC and TA (SSC:TA), flavor or external color (ExtCol). One moderate effect QTL for SSC was detected in the OR 2012 season on LG 6A and explained 10% of the total phenotypic variation (Table 1; Supplementary Figure S4). A minor effect QTL (2lnBF < 5) for FWT was detected on LG 2BII in the OR 2011 environment (Supplementary Figure S5). For pH, a major effect QTL was detected on LG 4CII (MI 2012; Table 1; Supplementary Figure S6), which explained 20% of the phenotypic variation. One moderate effect (5 ≤ 2lnBF < 10) TA QTL was detected on LG 2A in MI 2011 and a minor effect QTL for TA was detected on LG 5B in OR 2012 (Table 1; Supplementary Figure S7). The moderate effect TA QTLs on LG 2A explained 8% of the total phenotypic variation and the one on LG 5B explained 22% of the total phenotypic variation.

**Figure 2:**
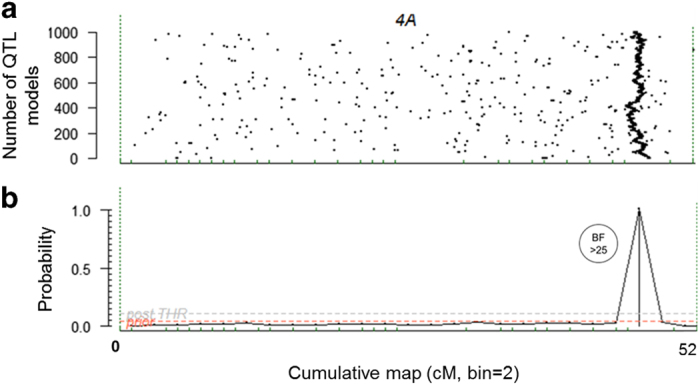
Posterior probability of PF QTL positions and traces of the QTL models along LG 4A for an additive genetic model. (**a**) Traces of number of QTL models: each black dot represents an independent QTL model and the consistent vertical line of black dots at the end of the chromosome indicates presence and location of the QTL, (**b**) posterior probability of QTL positions, BF=Bayes factor. BF > 25 is confirmation of a decisive evidence of presence of a QTL.

A single major effect QTL for PF was detected by genome-wide QTL analysis of haplotype block alleles (Table 1; Supplementary Figure S8) across both locations (MI and OR) and years (2011 and 2012). The PF QTL explained 68% and 33% of the total phenotypic variation in 2011 and 2012, respectively, at both locations and was detected on the distal end of LG 4A between 44.7 and 50.2 cM using SNP markers (Figure 2). Based on the founders used in this study, this QTL likely corresponds to the *FaPFRU* locus.^18,23,24^

**Figure 3:**
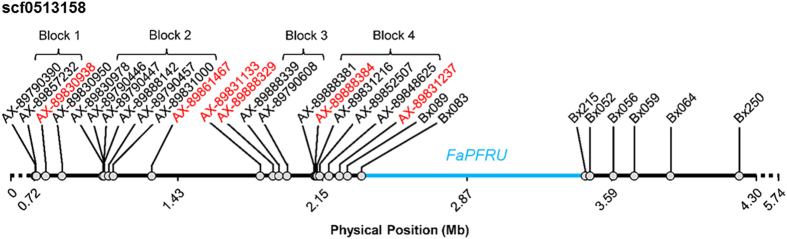
Physical positions of IStraw90 SNPs (AX prefix) and Perrotte and Gaston *et al.*^24^ SSR markers (Bx prefix) on *F. vesca* scaffold 0513158 associated with the flowering habit QTL *FaPFRU.* The 1.1 Mb region in blue delimits the *FaPFRU* locus as reported by Perrotte and Gaston *et al.* (2016a) and Gaston *et al.* (2013). Red markers were monomorphic in the MSU and ORUS breeding populations and Bx prefix markers were not evaluated in this study.

To confirm co-location of the PF QTL with *FaPFRU*, the sequences of 15 SNP markers associated with the QTL, and six additional SNP markers that were not used for the QTL analysis because they were either monomorphic or not high-quality markers within the QTL region, were BLAST searched against the scaffolds of the *F. vesca* v1.0 genome assembly. All markers aligned with scaffold 0513158 (Figure 3), which is the same scaffold previously identified as containing the *FaPFRU* region.^24^ To better visualize the *FaPFRU* region, these 21 SNP markers and the SSRs developed by Perrotte and Gaston *et al.*^24^ were physically mapped to scaffold 0513158. All of the SNP markers mapped proximally to the *FaPFRU* region defined by Perrotte and Gaston *et al.*^24^ (Figure 3).

### Assessment of EMFv006 marker for DNA informed breeding

The SSR marker EMFv006 linked to the 6A SSC QTL^12^ was used to screen the RosBREED strawberry population set. The SSR marker amplified five different alleles: 207bp, 209bp, 213bp, 215bp and 217bp. These five marker alleles constituted 12 different genotypes and phenotypic data were available for nine marker genotypes. Phenotypic distribution associated with each of the nine diplotypes were visualized by density plots and no clear separation of density curves were observed (Supplementary Figure S9). At the same time, phenotypic distribution associated with FlexQTL assigned *QQ*, *Qq*, *qq* diplotypes were also visualized and we observed separation in phenotypic distributions associated with *QQ* and *qq* with at least 50% overlapping region of the distributions (Supplementary Figure S9). This indicated that there might be significant association at the SNP level at the SSC locus on LG 6A. An analysis of variance model using these three QTL diplotypes (*QQ*, *Qq*, *qq*) explained approximately 20% of the total SSC variation within the strawberry breeding sets evaluated in OR 2012. However, when genetic screening of the RosBREED material was conducted with the SSR marker EMFv006, substantial homozygosity was observed. Further, high-resolution melting curve assay was performed to detect any sequence difference between the SSR marker EMFv006 homozygous alleles; however, no differences were detected. Therefore, EMFv006 was found to not be useful for MAS.

### Assessment of *FaPFRU* locus HBs and haplotypes for MAS

**Figure 4:**
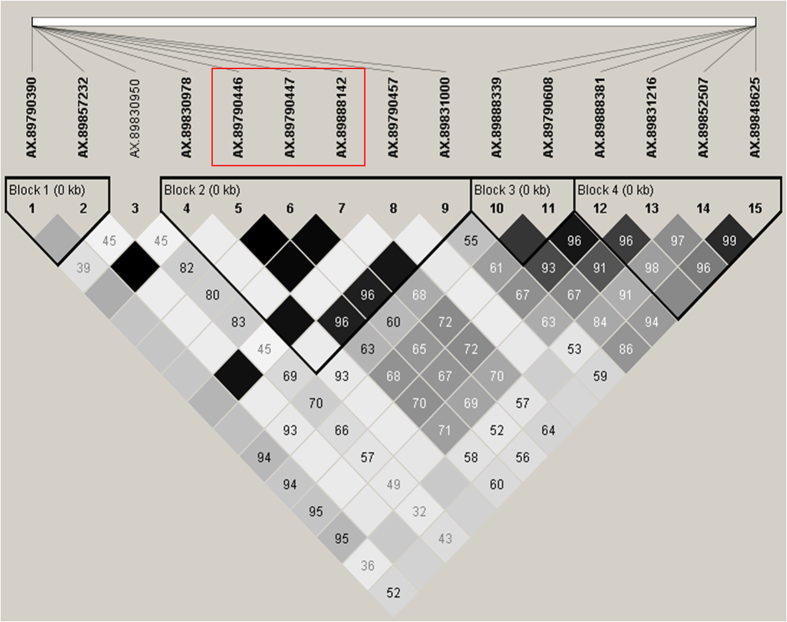
An overview of linkage disequilibrium (LD) (*r*^2^) and haplotype blocks for the genomic region containing 15 SNP markers associated with the perpetual/seasonal flowering QTL region. The three SNPs markers in the red box were used for haplotyping. The black triangular boxes represent HBs 1–4 from left to right. The (*r*^2^) values, from 1 to 0, are represented by dark black (higher) to gray colored square boxes (lower). The red box highlights the three SNP markers, in high LD, used for haplotyping.

Four HBs extending across the 15 SNPs associated with PF were identified within the germplasm using Haploview (Figures 3 and 4). A complex pattern of association of each of the 15 SNP marker emerged with each SNP marker within the QTL region having some level of predictive ability. Among the 15 SNPs associated with PF, 10 had relatively higher predictability for SF (negative predictive value > 0.95; Supplementary Figure S10. Individuals containing the BB diplotype for each of the 10 SNP markers were associated with SF, with a negative predictive value from 0.96 to 0.99. The three SNPs (AX-89790446, AX-89790447 and AX-89888142) within HB-2 were in high linkage disequillibrium (LD) with each other (Figure 4), and were predictive to SF, each with negative predictive values of 0.99. Of the seven remaining predictive SNP markers, the AA diplotypes of AX-89831000, AX-89790608 and AX-89888381 and the BB diplotypes of AX-89888339, AX-89831216, AX-89852507 and AX-89848625 were predictive for SF, with negative predictive values of at least 0.96 (Supplementary Fig. S10).

The three SNP markers of HB-2 were used jointly to haplotype the 249 seedlings and 11 parents from the 23 MSU and ORUS pedigree-linked populations. As these three SNP markers were in high LD, haplotype diversity was low. Four haplotypes were segregating in the unselected material (seedlings). Haplotype AAA was present in 29% of the seedlings, whereas haplotype BBB was found in 70% of the seedlings. Two other haplotypes, ABB and AAB, were rare (frequency ≤ 1%). Based on the PediHaplotyper prediction, the AAB haplotype is the result of a recombination between alleles originating within unidentified parents near the *FaPFRU* locus in families MSU_9–15 and MSU_9–16. One progeny, MSU_9-14-8, carried the rare haplotype ABB, which was inherited from recombination between haplotypes of ‘Seascape’ (mother) and an unidentified father. Among the most frequent haplotypes within unselected material, only four diplotypes (compound haplotype) were possible: AAA|AAA (4%), AAA|BBB (35%), BBB|AAA (14%) and BBB|BBB (45%).

**Figure 5:**
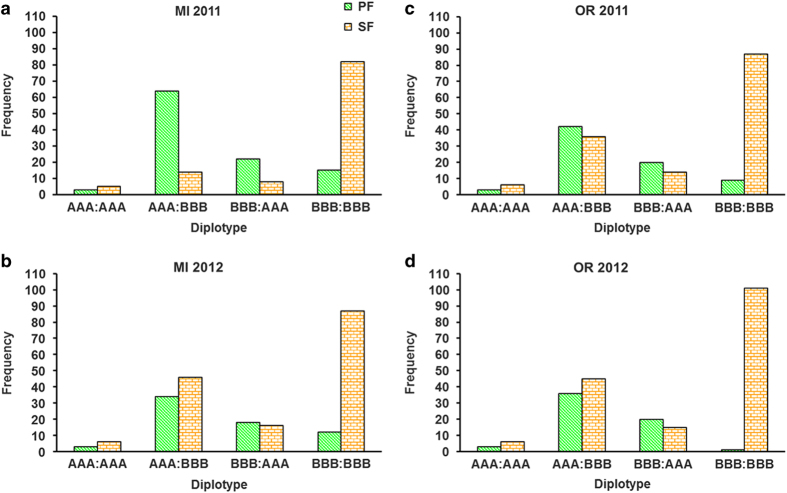
Association of diplotypes (three SNP markers in HB-2) with PF and SF octoploid strawberry (*Fragaria*×*ananassa*) in MI and OR over 2 years in (**a**) MI 2011, (**b**) MI 2012, (**c**) OR 2011 and (**d**) OR 2012.

The homozygous diplotype BBB|BBB was most predictive for SF as demonstrated by higher negative predictive values over positive predictive values in each environment (Figure 5; Table 2) and was most predictive of SF in OR 2012. Heterozygous and homozygous individuals carrying the AAA haplotype expressed mixed flowering behavior. The cultivars Fort Laramie, Seascape, Tribute and Fort Laramie 2 were heterozygous at the *FaPFRU* locus and PF, whereas ‘Puget Reliance’ was heterozygous at the *FaPFRU* locus and SF. However, ‘Earliglow’, ‘Totem’, ‘Honeoye’, ORUS 2427-1, ‘Mary’s Peak’, MSU 49 and MSU 56 were homozygous BBB|BBB and SF. Environmental variability appears to affect the diagnostic accuracy of HB-2, which ranged from 64 to 80.7% (Table 2).

## Discussion

In this study, numerous QTLs were identified for horticulturally important traits. Many of the QTLs identified using the PBA approach were also identified in other studies using the pseudo test cross strategy^3,11,12,14,15,23,24,26,27^ demonstrating this new methodology performs as good as previously established QTL analysis methods for strawberry. The PBA approach allowed for the QTLs to be evaluated in numerous genetic backgrounds, illustrating robust genotype-trait associations. In this study, we were able to clarify and pinpoint sub-genomic locations of QTLs. Moreover, the use of the 90K axiom array^31^ in the present study has allowed for the identification of significant QTLs with much higher resolution than in previous studies.

Correct pedigree information is critical for the PBA approach used and extreme care was taken to ensure correct pedigree information before conducting the QTL analysis. The power to detect a QTL using a PBA approach is affected by the frequency of the founder alleles in the population and is assessed via average allele representation (AAR). An AAR of 12.5 results in a 77.0%, 95.7%, 98.5% and 99.6% probability that each alternative allele is present at least 10, 8, 7 and 6 times in the population, respectively.^53^ An AAR of 12.5 units is recommended as the minimum for statistical power in representing the alleles of breeding parents. A minimum AAR of 12.5 can be achieved either by 23 progeny (0.5×23=11.5 AAR) and two parents (0.5×2= 1AAR) or by 25 progeny (0.5×25= 12.5 AAR) with unknown parents. There are other possibilities because of pedigree connections that can increase AAR because of relatedness as detailed by Peace *et al.*^53^ The AAR for the parents ORUS 2427-1, ‘Puget Reliance’ and ‘Totem’ was below the recommended threshold because of low numbers of true seedlings as compared with the rest of the parents. However, the alleles of the PF cultivars Seascape, Tribute and both genotypes of Fort Laramie, as well as the SF cultivar Earliglow, Honeoye, MSU 49 and MSU 56 were well represented with an AAR ranging between 12.5 in MSU 56 and 51.5 in ‘Tribute’ (Supplementary Information S1).

Although only minor (2lnBF < 5) and moderate (5 ≤ 2lnBF <10) QTLs were detected for four fruit quality traits, one major effect (2lnBF ≥ 10) QTL was detected for flowering habit. The moderate effect QTL for SSC detected on LG 6A is supportive of the SSC QTLs on LGs associated with chromosome 6 previously reported in QTL studies conducted in the cultivated strawberry thus far.^3,12,16^ However, owing to the low mapping densities and the type of markers used in previous studies, it is not clear that these previously reported QTLs belong to LG 6A or to homoeologous chromosomes. The remaining five fruit quality QTLs identified in this study coincided with QTLs reported by at least one other study for the same trait on the same HG chromosome. The FWT QTL we detected on LG 2BII was also observed on HG 2 by Lerceteau-Köhler *et al.*^12^ The pH QTL detected on LG 4CII in MI 2012 was also previously reported on HG 4, by Lerceteau-Köhler *et al.*^12^ and Zorrilla-Fontanesi *et al*.^3^ As with the SSC QTL on LG 6A, however, it cannot be confirmed that the other fruit quality QTLs reported here are in the same regions found in the earlier studies.

The SSC QTL on LG 6A explained 10% of total SSC variation in the breeding populations evaluated in OR 2012. The SSR marker EMFv006, which was previously reported by Lerceteau-Köhler *et al.*^12^ to be closely associated with the SSC QTL, was also found flanking the current SSC QTL (van de Weg E, unpublished data). The SSR marker EMFv006 alleles were not able to differentiate between QTL alleles assigned by FlexQTL and was rendered not suitable for MAB applications. To determine if unknown polymorphism existed within the SSR marker EMFv006, analysis by high-resolution melting and amplicon sequencing was performed and no polymorphism associated with SSC level was determined, supporting ineffectiveness of EMFv006 marker for MAS.

Interestingly, few QTLs of significance were detected for each of the fruit quality traits and many were not consistent across environments or years. Missing phenotypic data were not consistent across locations and years and this unbalanced representation of the phenotypic data might have hindered detection of fruit quality QTLs across locations and over years. In addition, many fruit quality traits have been shown to be quantitatively inherited and under large environmental influence.^3,12–15^ Environmental influences may have prevented the detection of some smaller effect QTLs, although QTLs may not have been detected because of two sources of ascertainment bias that are present in this study. First, regions of low mapping resolution will exist in areas of the ‘Holiday’×‘Korona’ map where both parents were homozygous. As genetic positions from the ‘Holiday’×‘Korona’ map were used for our analysis, this source of lower mapping resolution was transferred to this experiment. Second, artificial regions of homozygosity may have been created in the parents because of using only markers that are polymorphic in the ‘Holiday’×‘Korona’ population but not in the parental germplasm. In all likelihood, unobserved markers exist that are polymorphic between parental lines in these regions that could aid in the detection of QTLs, as well as assist in the phasing of alleles.

As many of the cultivars used in previous PF studies share ancestry with those used in this study, the major effect QTL for PF detected at the distal end of LG 4A is likely the *FaPFRU* locus.^11,15,16,18,23,24,26,27^ This is further supported by the mapping of all 21 SNP markers within the QTL region to *F. vesca* scaffold 0513158.^24^ High LD between SNP markers within HB-2 of the PF QTL region indicated co-inheritance of SNP marker haplotypes. HB-2 was approximately 1.5 Mb away from the *FaPFRU* locus and had significant association with flowering habit, with diplotype BBB|BBB being highly predictive of SF. Despite being highly associated with flowering, test accuracy varied among environments, ranging from 64 to 80.7%.

The genetic system that enables PF appears to be complex and likely constitutes several key genes and proteins that are capable of sensing environmental cues and hormone levels, which affect their function.^54^ This was reflected in the lack of clear 1:1 association of the putatively dominant AAA allele with PF in all families (Supplementary Table S3). We propose two possible reasons for this lack of clear association: (1) environmental conditions suppressed the expression of flowering related genes, and (2) the contribution of founder alleles whereby a recombination event or independent mutational events have caused the AAA haplotype to be linked in coupling to SF. OR and MI have very different environments, and fluctuations in temperature were an everyday phenomenon within these areas during the study period. Seasonal cues like temperature, light conditions and day length have a significant role in the floral transition leading to PF in strawberry.^55,56^ In addition, age-dependent vernalization also influences flowering.^57^ Perrotte and Gaston *et al.*^24^ and Salinas *et al.*^18^ identified SF cultivars, such as Earliglow and Elsanta, which had the diagnostic allele of Bx215 for PF demonstrating that recombination or mutational events have occurred in the region. As the SNP markers from HB-2 are physically more distant than Bx215 from *FaPFRU*, it is not unexpected for one of these events to have occurred.

In conclusion, by using multiple pedigrees, PBA determines the effect of alleles from multiple founders simultaneously. Ten QTLs combined were detected for fruit quality traits in MI and OR over 2 years and a major effect QTL was detected for flowering habit in all environments. The flowering QTL was detected in the same genomic region as that of *FaPFRU*. A haplotyping approach applied to the SNPs within the *FaPFRU* allowed us to achieve greater concordance with the flowering habits of germplasm in different environments. Alleles predictive of SF were identified and can be converted to a breeder-friendly DNA test to distinguish flowering habits in the cultivated strawberry. Future work will include further dissection of the genetic regions identified in order to better understand the molecular mechanisms driving these traits. This work will ultimately help breeders identify cultivars with desired traits and provide information to develop DNA-based markers for MAS.

## Figures and Tables

**Table 1:** Summary of QTLs detected in the 25 pedigree-linked strawberry (*Fragaria*×*ananassa*) populations phenotyped in Michigan and Oregon in 2011 and 2012

	*OR 2011*				*OR 2012*			
	*LGs*	*2lnBF*	*Interval (cM)*	*PVE (%)*	*LGs*	*2lnBF*	*Interval (cM)*	*PVE (%)*
SSC	—	—	—	—	6A	**6**	50–58	10
FWT	2BII	*4*	0–5	9	—	—	—	—
pH	—	—	—	—	—	—	—	—
TA	—	—	—	—	5B	**5**	1–4	10
						**5**	29–33	12
PF	4A	**31**	46–51	68	4A	**31**	47–50	33
	*MI 2011*				*MI 2012*			
SSC	—	—	—	—	—	—	—	—
FWT	—	—	—	—	—	—	—	—
pH	—	—	—	—	4CII	**9**	2–5	20
TA	2A	**7**	31–36	8	—	—	—	—
PF	4A	**31**	46–51	67	4A	**31**	47–50	33

Abbreviations: FWT, fruit weight; MI, Michigan; LG, linkage group; OR, Oregon; PVE, phenotypic variation explained; SSC, soluble solids content; TA, titratable acidity; PF, perpetual flowering.

Bayes factors ≥ 5 are highlighted as bold and Bayes factors ≤ 5 are italicized.

**Table 2:** Summary statistics of the diagnostic ability of HB-2 in four environments

*Environment*	N	P* value*	*Accuracy (%)*	*Positive predictive value (PF)*	*Negative predictive value (SF)*	*Sensitivity*	*Specificity*
MI 2011	212	< 2.2E-16	80.7	0.77	0.85	0.86	0.76
MI 2012	222	3.2E-07	64.0	0.45	0.88	0.82	0.56
OR 2011	217	0.0297	69.6	0.53	0.9	0.86	0.61
OR 2012	227	1.3E-14	70.5	0.47	0.99	0.98	0.6

Abbreviations: HB, haploblock; PF, perpetual flowering; MI, Michigan; OR, Oregon; SF, seasonal flowering.

The diagnostic test was interpreted as predicting SF if the diplotype is BBB|BBB. Diplotype association with flowering habit was assessed using Pearson’s chi-squared test of independence with Yates’s correction.

## References

[bib1] Whitaker VM. Applications of molecular markers in strawberry. J Berry Res 2011; 1: 115–127.

[bib2] Abbott JA. Quality measurement of fruits and vegetables. Postharvest Biol Technol 1999; 15: 207–225.

[bib3] Zorrilla-Fontanesi Y, Rambla J, Cabeza A et al. Genetic analysis of strawberry fruit aroma and identification of *O-Methyltransferase FaOMT* as the locus controlling natural variation in mesifurane content. Plant Physiol 2012; 159: 851–870. 22474217 10.1104/pp.111.188318 PMC3375946

[bib4] Chambers AH, Pillet J, Plotto A, Bai J, Whitaker VM, Folta KM. Identification of a strawberry flavor gene candidate using an integrated genetic-genomic-analytical chemistry approach. BMC Genomics 2014; 15: 217. 24742080 10.1186/1471-2164-15-217 PMC4023330

[bib5] Demmig-Adams B, Adams WW. Antioxidants in photosynthesis and human nutrition. Science 2002; 298: 2149–2153. 12481128 10.1126/science.1078002

[bib6] Battino M, Beekwilder J, Denoyes-Rothan B, Laimer M, McDougall GJ, Mezzetti B. Bioactive compounds in berries relevant to human health. Nutr Rev 2009; 67: 145–150. 10.1111/j.1753-4887.2009.00178.x 19453670

[bib7] Afrin S, Gasparrini M, Forbes-Hernandez TY et al. Promising health benefits of the strawberry: a focus on clinical studies. J Agric Food Chem 2016; 64: 4435–4449. 27172913 10.1021/acs.jafc.6b00857

[bib8] Schulenburg K, Feller A, Hoffmann T, Schecker JH, Martens S, Schwab W. Formation of β-glucogallin, the precursor of ellagic acid in strawberry and raspberry. J Exp Bot 2016; 67: 2299–2308. 26884604 10.1093/jxb/erw036 PMC4809288

[bib9] Song C, Ring L, Hoffmann T, Huang F, Slovin J, Schwab W. Acylphloglucinol biosynthesis in strawberry fruit. Plant Physiol 2015; 169: 1656–1670. 26169681 10.1104/pp.15.00794 PMC4634061

[bib10] Urrutia M, Schwab W, Hoffmann T, Monfort A. Genetic dissection of the (poly)phenol profile of diploid strawberry (*Fragaria vesca*) fruits using a NIL collection. Plant Sci 2016; 242: 151–168. 26566833 10.1016/j.plantsci.2015.07.019

[bib11] Rousseau-Gueutin M, Lerceteau-Köhler E, Barrot L et al. Comparative genetic mapping between octoploid and diploid *Fragaria* species reveals a high level of colinearity between their genomes and the essentially disomic behavior of the cultivated octoploid strawberry. Genetics 2008; 179: 2045–2060. 18660542 10.1534/genetics.107.083840 PMC2516079

[bib12] Lerceteau-Köhler E, Moing A, Guerin G et al. Genetic dissection of fruit quality traits in the octoploid cultivated strawberry high-lights the role of homoeo-QTL in their control. Theor Appl Genet 2012; 124: 1059–1077. 22215248 10.1007/s00122-011-1769-3 PMC3304055

[bib13] Schauer N, Semel Y, Balbo I et al. Mode of inheritance of primary metabolic traits in tomato. Plant Cell 2008; 20: 509–523. 18364465 10.1105/tpc.107.056523 PMC2329927

[bib14] Zorrilla-Fontanesi Y, Cabeza A, Domínguez P et al. Quantitative trait loci and underlying candidate genes controlling agronomical and fruit quality traits in octoploid strawberry (*Fragaria* ×*ananassa*). Theor Appl Genet 2011; 123: 755–778. 21667037 10.1007/s00122-011-1624-6

[bib15] Castro P, Lewers KS. Identification of quantitative trait loci (QTL) for fruit-quality traits and number of weeks of flowering in the cultivated strawberry. Mol Breed 2016; 36: 138.

[bib16] Castro P, Bushakra JM, Stewart P et al. Genetic mapping of day-neutrality in cultivated strawberry. Mol Breed 2015; 35: 79.

[bib17] Heide OM, Stavang JA, Sønsteby A. Physiology and genetics of flowering in cultivated and wild strawberries-A review. J Hort Sci Biotechnol 2013; 88: 1–18.

[bib18] Salinas NR, Zurn JD, Mathey M et al. Validation of molecular markers associated with perpetual flowering in octoploid *Fragaria* germplasm. Mol Breed 2017; 37: 70.

[bib19] Darrow GM. The Strawberry History, Breeding and Physiology Holt. Rinehart and Winston: New York, NY, USA. 1996.

[bib20] Hancock JF. Fruiting and Postharvest Physiology Strawberries. CABI Publishing: Wallingford, UK. 1999.

[bib21] Bringhurst RS, Voth V. Six new strawberry varieties released. Calif Agr 1980; 34: 12–15.

[bib22] Hancock JF. Strawberries In: Erez A (ed). Temperate Fruit Crops in Warm Climates. Springer: Dordrecht, The Netherlands. 2000, pp 445–455.

[bib23] Gaston A, Perrotte J, Lerceteau-Kohler E et al. *PFRU*, a single dominant locus regulates the balance between sexual and asexual plant reproduction in cultivated strawberry. J Exp Bot 2013; 64: 1837–1848. 23554259 10.1093/jxb/ert047

[bib24] Perrotte J, Gaston A, Potier A, Petit A, Rothan C, Denoyes B. Narrowing down the single homoeologous *FaPFRU* locus controlling flowering in cultivated octoploid strawberry using a selective mapping strategy. Plant Biotechnol J 2016a; 14: 2176–2189. 27168086 10.1111/pbi.12574 PMC5095798

[bib25] Perrotte J, Guédon Y, Gaston A, Denoyes B. Identification of successive flowering phases highlights a new genetic control of the flowering pattern in strawberry. J Exp Bot 2016b; 67: 5643–5655. 27664957 10.1093/jxb/erw326 PMC5066487

[bib26] Sooriyapathirana SS, Mookerjee S, Weebadde CK et al. Identification of QTL associated with flower and runner production in octoploid strawberry (*Fragaria* ×*ananassa*). J Berry Res 2015; 5: 107–116.

[bib27] Weebadde CK, Wang D, Finn CE et al. Using a linkage mapping approach to identify QTL for day-neutrality in the octoploid strawberry. Plant Breed 2008; 127: 94–101.

[bib28] Honjo M, Nunome T, Kataoka S et al. Simple sequence repeat markers linked to the everbearing flowering gene in long-day and day-neutral cultivars of the octoploid cultivated strawberry *Fragaria ×ananassa*. Euphytica 2016; 209: 291–303.

[bib29] Bink MCAM, Jansen J, Madduri M et al. Bayesian QTL analyses using pedigreed families of an outcrossing species, with application to fruit firmness in apple. Theor Appl Genet 2014; 127: 1073–1090. 24567047 10.1007/s00122-014-2281-3

[bib30] van de Weg WE, Voorrips RE, Finker R, Kodde LP, Meulenbroek EJ. Pedigree genotyping: a new pedigree-based approach of QTL identification and allele mining by exploiting breeding material. Acta Hortic 2006; 708: 483–488.

[bib31] Bassil NV, Davis TM, Zhang H et al. Development and preliminary evaluation of a 90 K Axiom® SNP array for the allo-octoploid cultivated strawberry *Fragaria* ×*ananassa*. BMC Genomics 2015; 16: 155. 25886969 10.1186/s12864-015-1310-1 PMC4374422

[bib32] Roach JA, Verma S, Peres NA et al. *FaRXf1*: a locus conferring resistance to angular leaf spot caused by *Xanthomonas fragariae* in octoploid strawberry. Theor Appl Genet 2016; 6: 1191–1201. 10.1007/s00122-016-2695-1 26910360

[bib33] Mangandi J, Verma S, Osorio L, Peres NA, Van de Weg E, Whitaker VM. Pedigree-based analysis in a multiparental population of octoploid strawberry reveals QTL alleles conferring resistance to *Phytophthora cactorum*. G3 2017; 7: 1707–1719. 28592652 10.1534/g3.117.042119 PMC5473751

[bib34] Voorrips RE, Bink MCAM, Kruisselbrink JW, Koehorst-van Putten HJJ, van de Weg WE. PediHaplotyper: software for consistent assignment of marker haplotypes in pedigrees. Mol Breed 2016; 36: 119. 27547106 10.1007/s11032-016-0539-y PMC4977329

[bib35] Mathey MM, Mookerjee S, Gündüz K et al. Largescale standardized phenotyping of strawberry in RosBREED. J Am Pomol Soc 2013; 67: 205–216.

[bib36] R Core Team. R: A language and environment for statistical computing R Foundation for Statistical Computing, Vienna, 2016, Austria http://wwwR-projectorg/.

[bib37] Gilmore BS, Bassil NV, Hummer KE. DNA extraction protocols from dormant buds of twelve woody plant genera. J Am Pomolog Soc 2011; 65: 201–206.

[bib38] Voorrips RE, Bink MCAM, van de Weg WE. Pedimap: software for the visualization of genetic and phenotypic data in pedigrees. J Hered 2012; 103: 903–907. 23087384 10.1093/jhered/ess060 PMC3510005

[bib39] Van Dijk T, Pagliarani G, Pikunova A et al. Genomic rearrangements and signatures of breeding in the allo-octoploid strawberry as revealed through an allele dose based SSR linkage map. BMC Plant Biol 2014; 14: 55. 24581289 10.1186/1471-2229-14-55 PMC3944823

[bib40] Rosyara UR, Bink MCAM, van de Weg E et al. Fruit size QTL identification and the prediction of parental QTL genotypes and breeding values in multiple pedigreed populations of sweet cherry. Mol Breed 2013; 32: 875–887.

[bib41] Fresnedo-Ramírez J, Bink MCAM, van de Weg E et al. QTL mapping of pomological traits in peach and related species breeding germplasm. Mol Breed 2015; 35: 166.

[bib42] Guan Y, Peace C, Rudell D, Verma S, Evans K. QTLs detected for individual sugars and soluble solids content in apple. Mol Breed 2015; 35: 135.

[bib43] Allard A, Bink MC, Martinez S et al. Detecting QTLs and putative candidate genes involved in budbreak and flowering time in an apple multiparental population. J Exp Bot 2016; 67: 2875–2888. 27034326 10.1093/jxb/erw130 PMC4861029

[bib44] Voorrips RE. MapChart: software for the graphical presentation of linkage maps and QTLs. J Hered 2002; 93: 77–77. 12011185 10.1093/jhered/93.1.77

[bib45] Barrett JC, Fry B, Maller J, Daly MJ. Haploview: analysis and visualization of LD and haplotype maps. Bioinformatics 2005; 21: 263–265. 15297300 10.1093/bioinformatics/bth457

[bib46] Shulaev V, Sargent DJ, Crowhurst RN et al. The genome of woodland strawberry (*Fragaria vesca*). Nat Genet 2010; 43: 109–116. 21186353 10.1038/ng.740 PMC3326587

[bib47] Langmead B, Trapnell C, Pop M, Salzberg SL. Ultrafast and memory-efficient alignments of short DNA sequences to the human genome. Genome Biol 2009; 10: R25. 19261174 10.1186/gb-2009-10-3-r25 PMC2690996

[bib48] Li H, Handsaker B, Wysoker A et al. 1000 Genome Project Data Processing Subgroup. The sequence alignment/map format and SAMtools. Bioinformatics 2009; 25: 2078–2079. 19505943 10.1093/bioinformatics/btp352 PMC2723002

[bib49] Robinson JT, Thorvaldsdóttir H, Winckler W et al. Integrative genomics viewer. Nat Biotechnol 2011; 29: 24–26. 21221095 10.1038/nbt.1754 PMC3346182

[bib50] Thorvaldsdóttir H, Robinson JT, Mesirov JP. Integrative genomics viewer (IGV): high-performance genomics data visualization and exploration. Brief Bioinform 2013; 14: 178–192. 22517427 10.1093/bib/bbs017 PMC3603213

[bib51] Aliu O, Chung KC. Assessing strength of evidence in diagnostic tests. Plast Reconstr Surg 2012; 129: 989e–998e. 10.1097/PRS.0b013e31824ecd61 PMC3361685 22634696

[bib52] Glas AS, Lijimer JG, Prins MH, Bonsel GJ. Bossuyt PMM. The diagnostic odds ratio: a single indicator of test performance. J Clin Epidemiol 2003; 56: 1129–1135. 14615004 10.1016/s0895-4356(03)00177-x

[bib53] Peace CP, Luby JJ, van de Weg WE, Bink MCAM, Iezzoni AF. A strategy for developing representative germplasm sets for systematic QTL validation, demonstrated for apple, peach, and sweet cherry. Tree Genet Genomes 2014; 10: 1679–1694.

[bib54] Andrés F, Coupland G. The genetic basis of flowering responses to seasonal cues. Nat Rev Genet 2012; 13: 627–639. 22898651 10.1038/nrg3291

[bib55] Koskela EA, Mouhu K, Albani MC et al. Mutation in *TERMINAL FLOWER1* reverses the photoperiodic requirement for flowering in the wild strawberry *Fragaria vesca*. Plant Physiol 2012; 159: 1043–1054. 22566495 10.1104/pp.112.196659 PMC3387692

[bib56] Nakano Y, Higuchi Y, Yoshida Y, Hisamatsu T. Environmental responses of the *FT*/*TFL1* gene family and their involvement in flower induction in *Fragaria* ×*ananassa*. J Plant Physiol 2015; 177: 60–66. 25666540 10.1016/j.jplph.2015.01.007

[bib57] Romera-Branchat M, Andrés F, Coupland G. Flowering responses to seasonal cues: what's new? Curr Opin Plant Biol 2014; 21: 120–127. 25072635 10.1016/j.pbi.2014.07.006

