# Supplementary material for: Clarifying sub-genomic positions of QTLs for flowering habit and fruit quality in U.S. strawberry (Fragaria×ananassa) breeding populations using pedigree-based QTL analysis
Source: Hortic Res. 2017 Nov 8;4:17062–. doi: 10.1038/hortres.2017.62 (PMC5676184; doi:10.1038/hortres.2017.62)
Supplement: Supplementary Information S1 [file hortres201762-s1.docx]

Clarifying sub-genomic positions of QTLs for flowering habit and fruit quality in U.S. strawberry (*Fragaria* ×*ananassa*) breeding populations using pedigree-based QTL analysis

**Running Title**: Strawberry flowering and fruit quality QTL

**Running Title**: Strawberry flowering and fruit quality QTL

**Name(s) of the authors(s):** Sujeet Verma, Jason D. Zurn, Natalia Salinas, Megan M. Mathey, Beatrice Denoyes, James F. Hancock, Chad E. Finn, Nahla V. Bassil, and Vance M. Whitaker

This supplementary materials file totaling fifteen pages contains three supplementary tables, S1-S3, followed by nine supplementary figures, S1-S10.

**SUPPLEMENTARY TABLES**

**Supplementary Table S1.** Summary statistics of phenotypic data for the eight fruit quality traits (fruit weight (g/fruit), FWT; pH; soluble solids content (%), SSC; titratable acidity, TA (g/L); external color, ExtCol; firmness, FIRM; and SSC:TA ratio) in Oregon and Michigan in 2011 and 2012. s.dev= standard deviation; n=available phenotypic data, NA= missing phenotypic data.

|  | **Min** | **Max** | **Mean** | **s. dev** | **n** | **NA** |
| --- | --- | --- | --- | --- | --- | --- |
| MI2011FWT | 0.50 | 18.54 | 5.41 | 2.95 | 102 | 192 |
| MI2012FWT | 1.30 | 21.80 | 7.78 | 3.27 | 242 | 52 |
| OR2011FWT | 4.32 | 37.60 | 16.94 | 6.41 | 219 | 75 |
| OR2012FWT | 1.16 | 29.30 | 9.42 | 4.26 | 167 | 127 |
| MI2011pH | 3.30 | 4.00 | 3.55 | 0.14 | 97 | 197 |
| MI2012pH | 3.20 | 3.94 | 3.48 | 0.14 | 239 | 55 |
| OR2011pH | 3.07 | 4.13 | 3.46 | 0.15 | 228 | 66 |
| OR2012pH | 3.01 | 3.87 | 3.34 | 0.14 | 166 | 128 |
| MI2011SSC | 7.00 | 18.70 | 11.37 | 2.21 | 102 | 192 |
| MI2012SSC | 6.20 | 15.90 | 10.28 | 1.99 | 241 | 53 |
| OR2011SSC | 3.10 | 14.70 | 8.29 | 1.52 | 227 | 67 |
| OR2012SSC | 3.17 | 10.80 | 7.13 | 1.18 | 166 | 128 |
| MI2011TA | 0.46 | 1.34 | 0.89 | 0.20 | 97 | 197 |
| MI2012TA | 0.58 | 1.88 | 1.10 | 0.25 | 241 | 53 |
| OR2011TA | 0.29 | 1.46 | 0.81 | 0.20 | 220 | 74 |
| OR2012TA | 0.37 | 1.71 | 0.69 | 0.18 | 166 | 128 |
| MI2011ExtCol | 3 | 9 | 7.51 | 1.05 | 105 | 189 |
| MI2012ExtCol | 4 | 9 | 6.63 | 0.92 | 242 | 52 |
| OR2011ExtCol | 4 | 9 | 7.27 | 0.82 | 227 | 67 |
| OR2012ExtCol | 5 | 8 | 6.64 | 0.74 | 85 | 209 |
| MI2011FIRM | 3 | 9 | 7.22 | 0.97 | 106 | 188 |
| MI2012FIRM | 2 | 9 | 5.36 | 1.41 | 243 | 51 |
| OR2011FIRM | 1 | 8 | 4.39 | 1.76 | 227 | 67 |
| OR2012FIRM | 3 | 8 | 5.66 | 1.18 | 86 | 208 |
| MI2011SSC:TA | 6.85 | 26.44 | 13.31 | 3.94 | 97 | 197 |
| MI2012SSC:TA | 4.93 | 21.85 | 9.71 | 2.65 | 241 | 53 |
| OR2011SSC:TA | 4.59 | 35.06 | 10.93 | 3.55 | 205 | 89 |
| OR2012SSC:TA | 3.53 | 19.16 | 10.79 | 2.75 | 166 | 128 |

**Supplementary Table S2.** Summary statistics of the genetic map used for QTL detection in this analysis.

| **Linkage group** | **Subgenome** | **# markers** | **Length (cM)** | **Maximum distance (cM)** |
| --- | --- | --- | --- | --- |
| 1 | 1A | 529 | 54.4 | 3.3 |
| 2 | 1B | 374 | 65.6 | 4.2 |
| 3 | 1C | 202 | 27.7 | 4.9 |
| 3 | 1CII | 21 | 1.4 | 1.4 |
| 4 | 1D | 86 | 11.6 | 2.2 |
| 4 | 1DII | 25 | 1.5 | 1.5 |
| 5 | 2A | 430 | 84.4 | 9.2 |
| 6 | 2B | 244 | 56.8 | 4.8 |
| 6 | 2BII | 71 | 10.8 | 2.7 |
| 7 | 2C | 113 | 21.0 | 4.2 |
| 7 | 2CII | 183 | 30.0 | 10.3 |
| 8 | 2D | 306 | 73.5 | 6.2 |
| 9 | 3A | 467 | 72.7 | 7.5 |
| 9 | 3AII | 82 | 15.0 | 4.2 |
| 10 | 3B | 437 | 60.6 | 4.4 |
| 11 | 3C | 467 | 79.6 | 4.8 |
| 12 | 3D | 312 | 82.7 | 8.5 |
| 13 | 4A | 327 | 47.0 | 4.1 |
| 13 | 4AII | 49 | 1.3 | 1.3 |
| 14 | 4B | 412 | 75.5 | 13.7 |
| 15 | 4C | 78 | 37.5 | 10.2 |
| 15 | 4CII | 31 | 5.6 | 4.2 |
| 16 | 4D | 327 | 79.9 | 11.1 |
| 17 | 5A | 586 | 74.9 | 7.0 |
| 18 | 5B | 458 | 60.2 | 5.5 |
| 18 | 5BII | 14 | 0.0 | 0.0 |
| 19 | 5C | 320 | 65.6 | 4.6 |
| 19 | 5CII | 30 | 4.1 | 1.4 |
| 20 | 5D | 337 | 64.1 | 5.6 |
| 21 | 6A | 677 | 91.8 | 5.7 |
| 21 | 6AII | 24 | 12.3 | 5.3 |
| 22 | 6B | 518 | 104.0 | 5.9 |
| 23 | 6C | 472 | 128.7 | 18.1 |
| 24 | 6D | 546 | 90.8 | 4.2 |
| 25 | 7A | 331 | 74.4 | 24.5 |
| 26 | 7B | 288 | 73.2 | 17.6 |
| 27 | 7C | 138 | 4.7 | 1.4 |
| 28 | 7D | 248 | 66.6 | 12.5 |
|  | **1A-7D** | **10560** | **1911.4** | **24.5** |

**Supplementary Table S3.** Expected and observed segregation of perpetual flowering (PF) and seasonal flowering (SF) traits in F_1_ individuals. Ratios for phenotypic observations are expressed as the number of PF individuals to the number of SF individuals. Parental germplasm in bold fonts are heterozygous, and italicized families have one unknown parent. No information are presented for families with progeny numbers less than 6.

| **Family** | **Parent1** | **Parent2** | **# progeny** | **Expected genetic segregation** | **Observed genetic**  **segregation** | **Observed**  **phenotype**  **in MI 2011** | **Observed**  **phenotype**  **in OR 2011** | **Observed**  **phenotype**  **in MI 2012** | **Observed**  **phenotype**  **in MI 2012** |
| --- | --- | --- | --- | --- | --- | --- | --- | --- | --- |
| MSU 9-10 | **Fort Laramie** | MSU 49 | 10 | 1:1 | 1:1 | 5:5 | 1:9 | 3:7 | 2:8 |
| MSU 9-11 | **Fort Laramie** | MSU 56 | 7 | 1:1 | - | 5:2 | 2:5 | 1:6 | 3:4 |
| MSU 9-13 | **Seascape** | Honeoye | 6 | 1:1 | 1:1 | 1:5 | 0:6 | 0:6 | 0:6 |
| MSU 9-14 | **Seascape** | MSU 56 | 7 | 1:1 | 1:1 | 4:2 | 3:4 | 1:6 | 3:4 |
| MSU 9-15 | **Tribute** | Earliglow | 9 | 1:1 | 1:1 | 7:1 | 3:5 | 4:4 | 4:4 |
| MSU 9-16 | **Tribute** | Honeoye | 53 | 1:1 | 1:1 | 29:21 | 21:28 | 23:29 | 13:40 |
| MSU 9-17 | **Tribute** | MSU 56 | 8 | 1:1 | 1:1 | 4:4 | 4:4 | 1:7 | 3:5 |
| *MSU 9-18* | **Seascape** | **F_MSU 9-18** | 10 | 1:2:1 | 1:2:1 | 3:6 | 4:5 | 4:6 | 5:5 |
| MSU 9-3 | MSU 49 | **Tribute** | *4* | - | - | - | - | - | - |
| MSU 9-2 | MSU 49 | Seascape | 10 | 1:1 | 1:1 | 3:3 | 4:5 | 1:5 | 4:5 |
| MSU 9-6 | Earliglow | Seascape | *3* | - | - | - | - | - | - |
| MSU 9-8 | Earliglow | **Fort Laramie** | 10 | 1:1 | 1:1 | 5:5 | 5:5 | 5:5 | 4:6 |
| MSU 9-9 | **Fort Laramie** | Honeoye | 10 | 1:1 | 1:1 | 5:5 | 2:8 | 1:9 | 1:9 |
| ORUS 3278 | ORUS 2427-1 | Seascape | 9 | 1:1 | 1:1 | 2:6 | 2:7 | 2:7 | 1:8 |
| ORUS 3279 | ORUS 2427-1 | **Tribute** | 9 | 1:1 | 1:1 | 7:2 | 3:6 | 3:6 | 1:8 |
| *ORUS 3305* | M_ORUS 3305 | **Seascape** | 10 | - | 1:1 | 5:4 | 3:6 | 2:7 | 2:7 |
| ORUS 3315 | **Fort Laramie_2** | **Puget Reliance** | 9 | 1:2:1 | 1:2:1 | 0:10 | 0:9 | 0:10 | 0:10 |
| ORUS 3316 | **Fort Laramie_2** | Totem | 8 | 1:1 | 1:1 | 0:7 | 0:5 | 1:7 | 0:8 |
| *ORUS 3320* | M_ORUS 3320 | Totem | 10 | - | - | 7:0 | 9:0 | 6:3 | 8:1 |
| ORUS 3323 | **Seascape** | **Puget Reliance** | 6 | 1:2:1 | - | 2:4 | 2:4 | 1:5 | 1:5 |
| *ORUS 3324* | M_ORUS 3324 | **Fort Laramie_2** | 10 | - | 1:1 | 9:0 | 9:0 | 1:9 | 0:10 |
| *ORUS 3325* | M_ORUS 3325 | Tribute | 10 | - | - | 2:8 | 2:8 | 1:9 | 1:9 |
| ORUS 3326 | **Tribute** | **Puget Reliance** | 6 | 1:2:1 | 1:2:1 | 2:3 | 2:3 | 2:3 | 1:4 |

**SUPPLEMENTARY FIGURES**


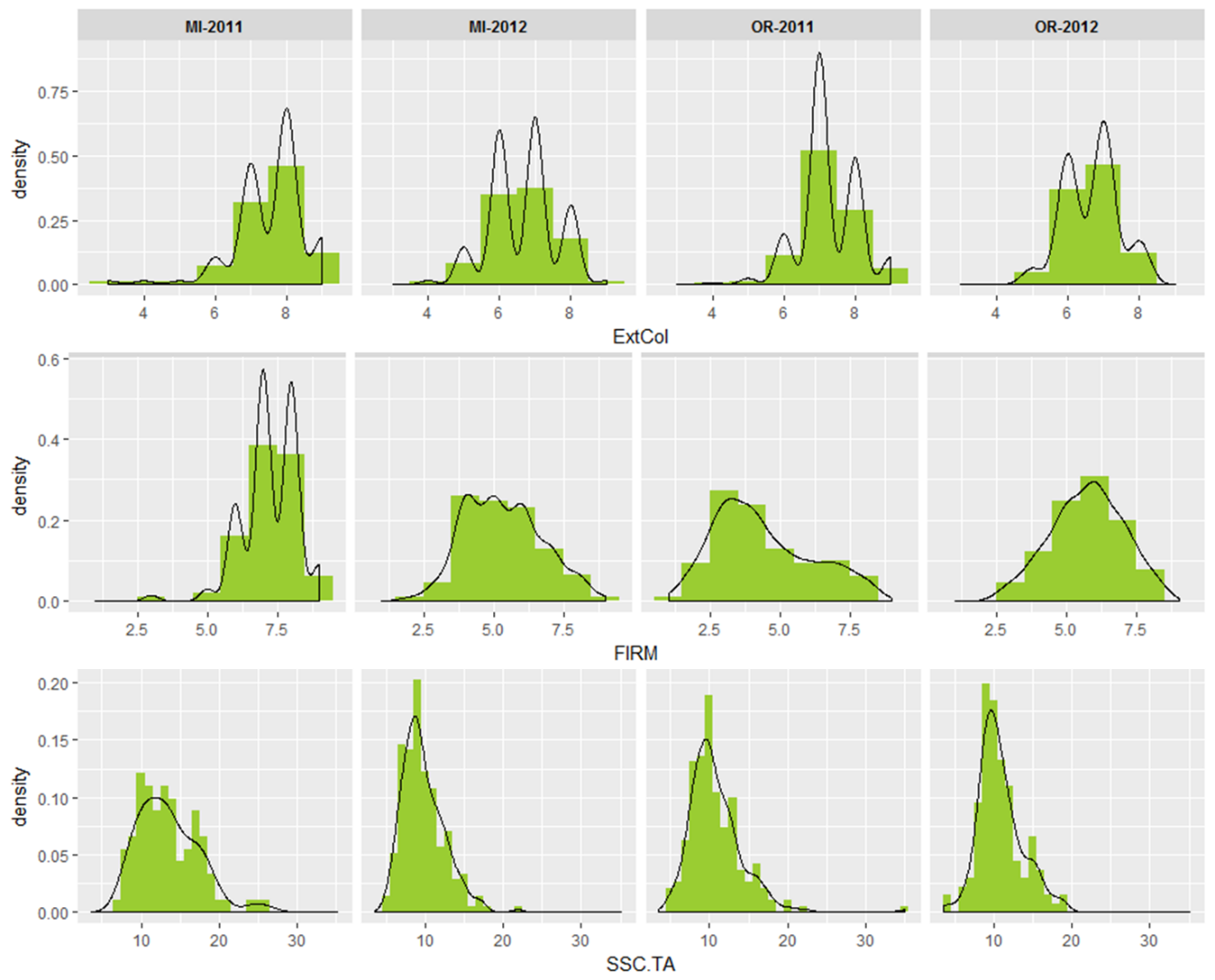


**Supplementary Figure S1.** Density plots of external color (ExtCol), firmness (FIRM), and ratio of SSC:TA (SSC:TA) for Michigan (MI) and Oregon (OR) in 2011 and 2012. Density fit lines are indicated by black lines.


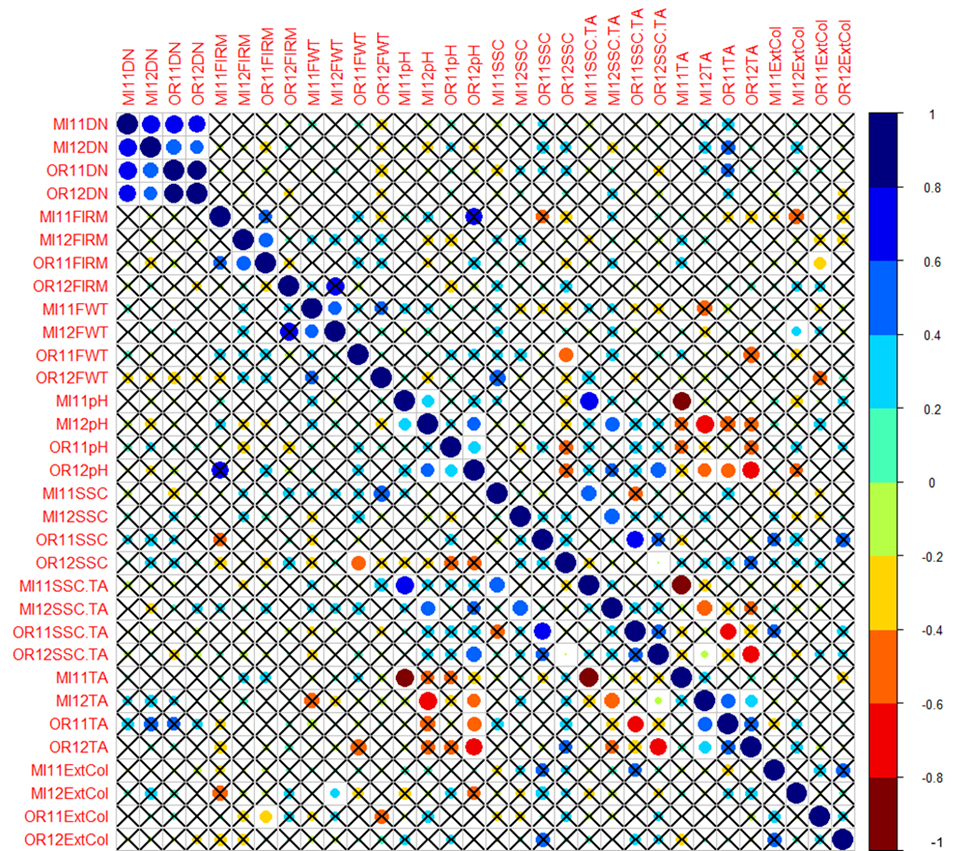


**Supplementary Figure 2.** Heatmap of Spearman’s rank correlation coefficients (r) among traits. P-values were adjusted using Bonferroni correction method for multiple comparisons. Cross symbol represents correlations coefficients not significant (α = 0.05). DN = perpetual flowering, FIRM = firmness, FWT = fruit weight; pH, SSC = soluble solids content, SSC:TA = ratio of SSC vs TA, TA = titratable acidity, and ExtCol = external color.


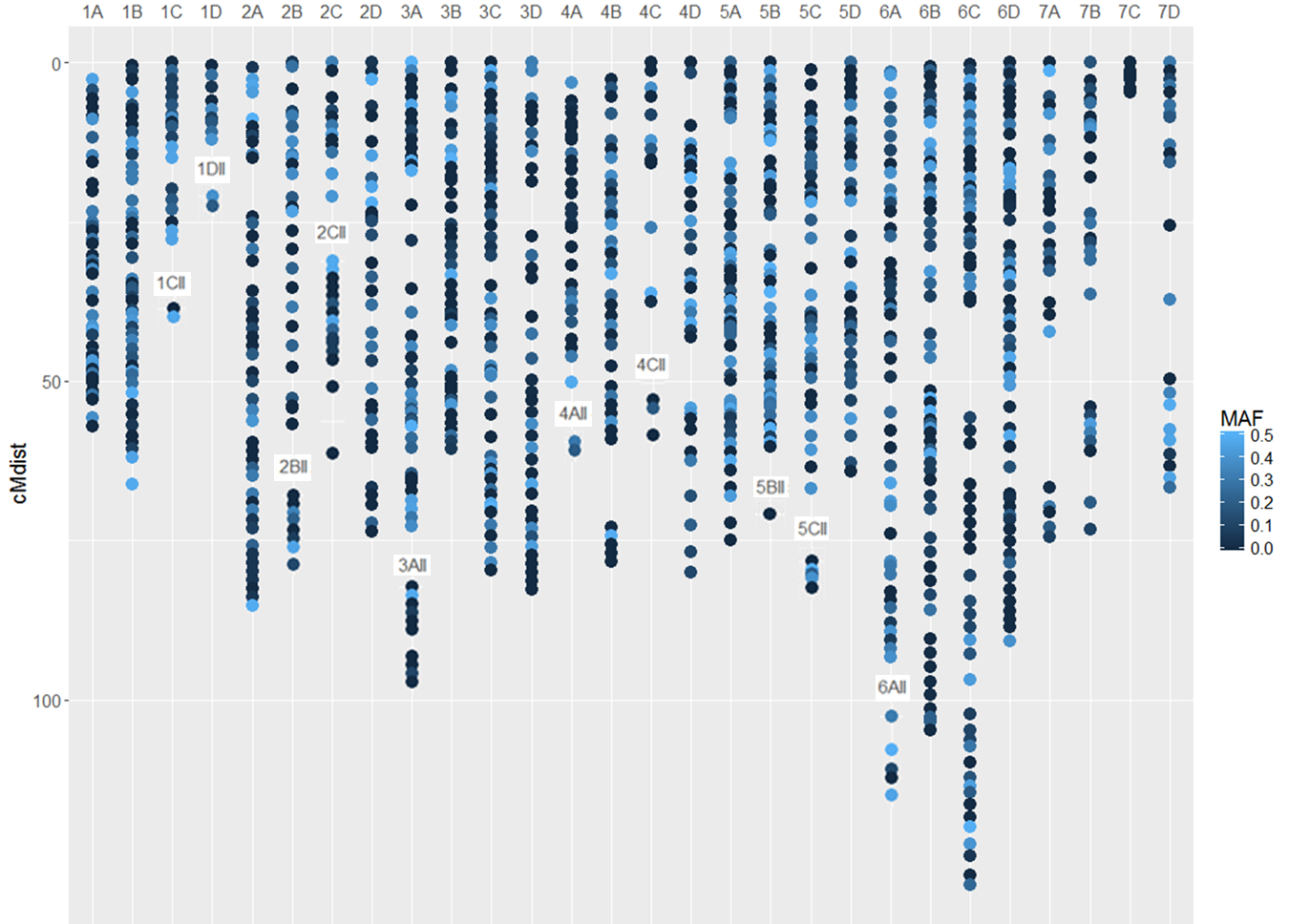


**Supplementary Figure S3.** Sub-genome wise distribution of minor allele frequency (MAF) in the breeding germplasm set. X-axis represents subgenomes and Y-axis represents genetic distances in cM.


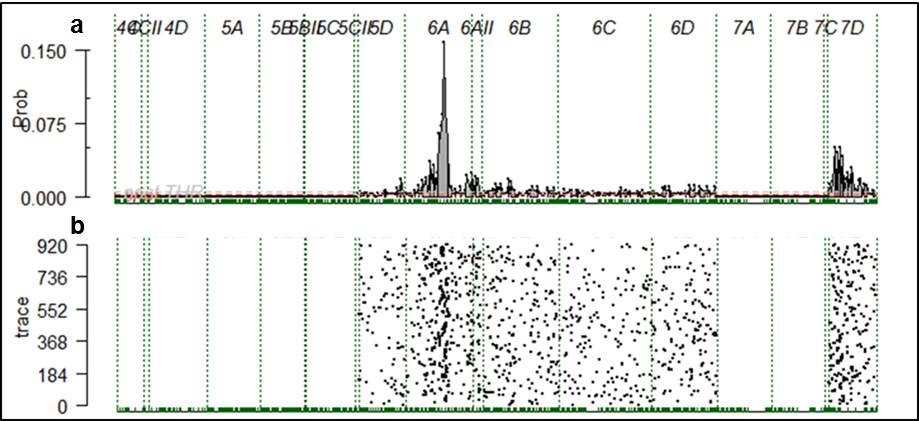


**Supplementary Figure S4.** SSC QTLs detected using the OR 2012 dataset. X-axis represents subgenomes. **(a)** posterior probability plot, and **(b)** traces of QTL models.


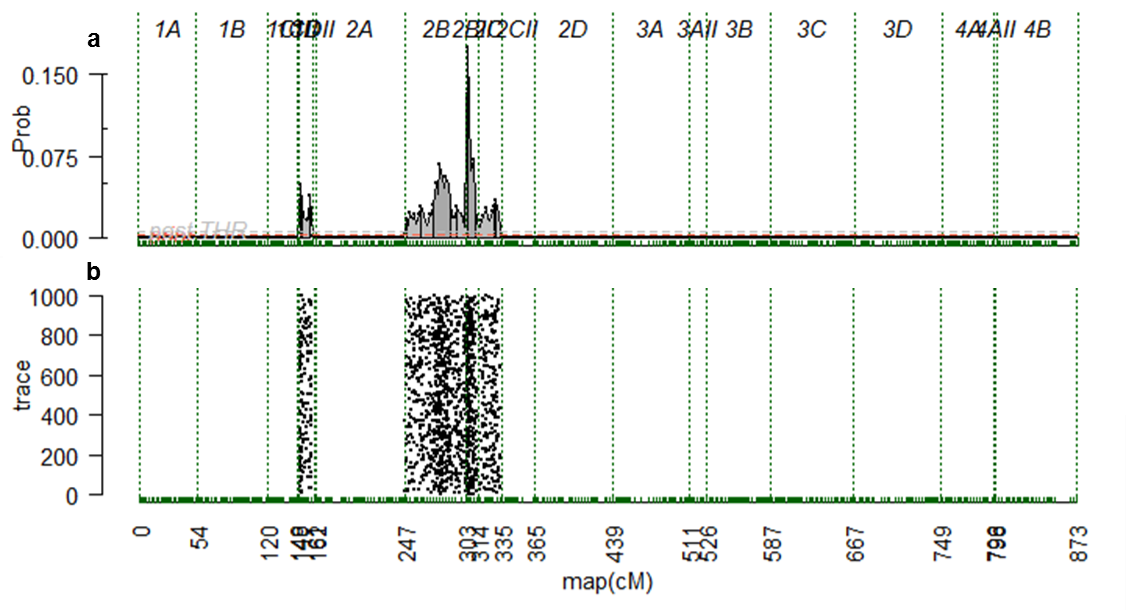


**Supplementary Figure S5.** FWT QTLs detected using the OR 2011 dataset. X -axis represents subgenomes. **(a)** posterior probability plot, and **(b)** traces of QTL models.

**
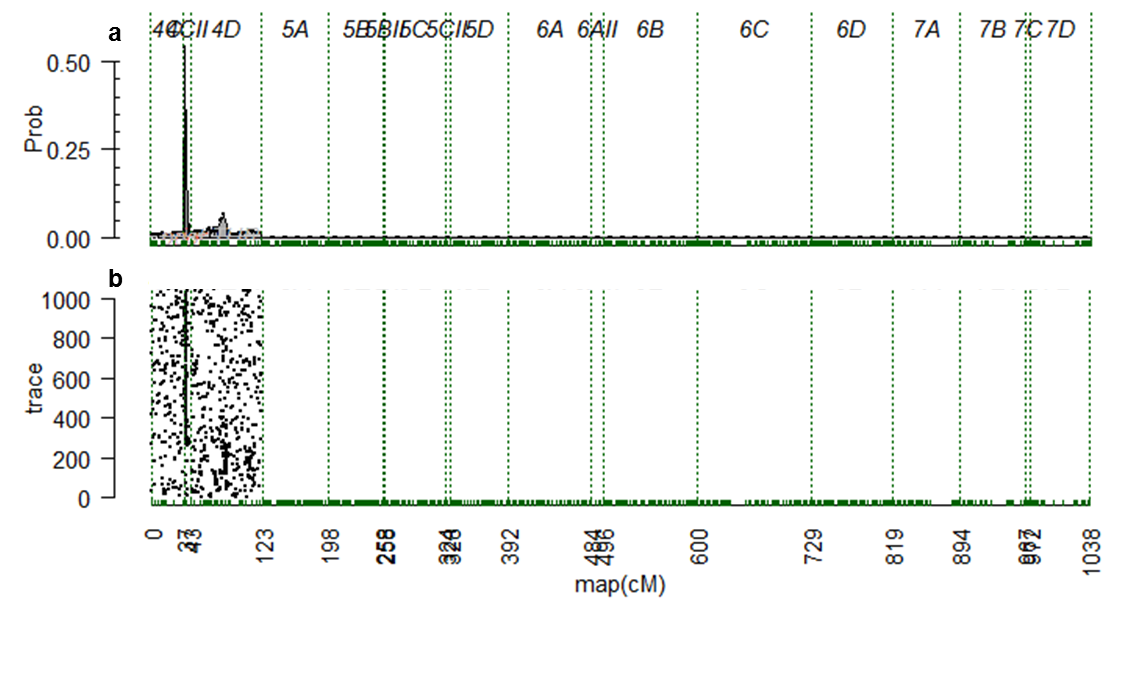
**

**Supplementary Figure S6.** pH QTLs detected using the MI 2012 dataset. **(a)** posterior probability plot, and **(b)** traces of QTL models.

**
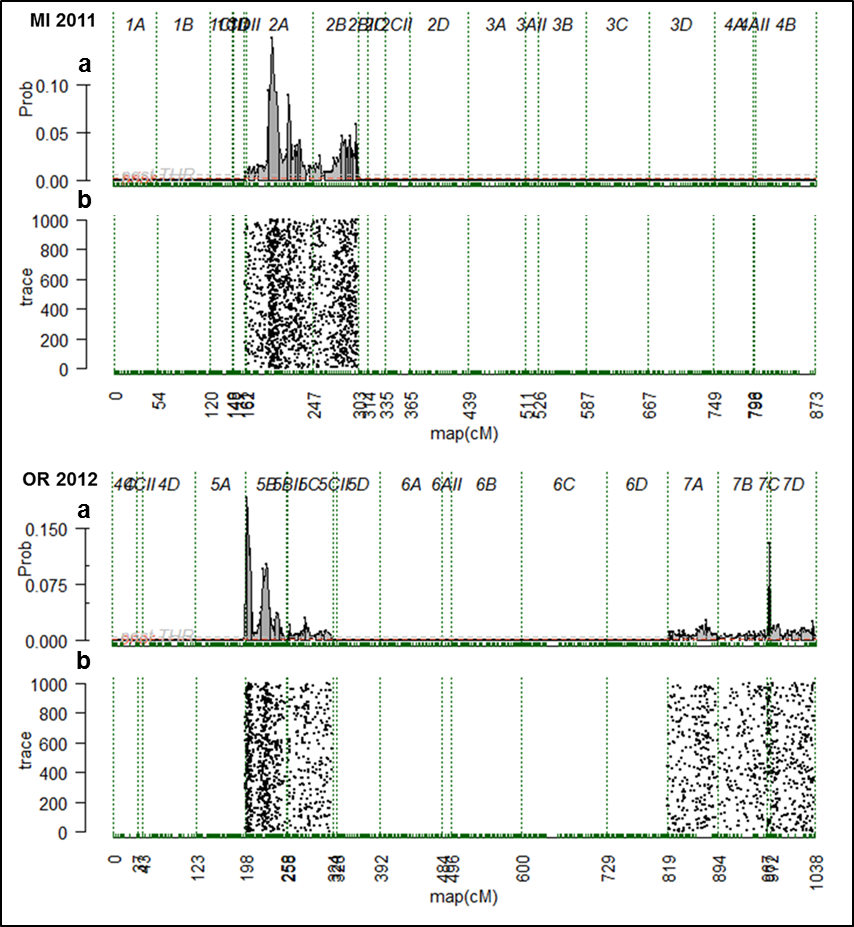
**

**Supplementary Figure S7.** TA QTLs detected using the MI 2011 and OR 2012 dataset. X -axis represents subgenomes. **(a)** posterior probability plot, and **(b)** traces of QTL models.


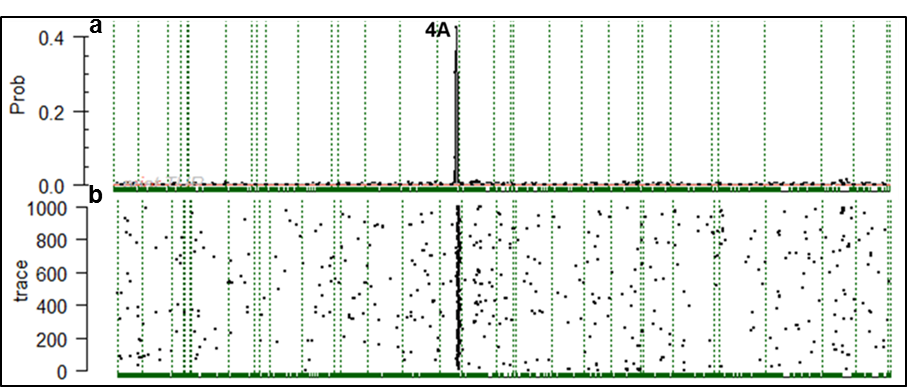


**Supplementary Figure S8.** QTL analysis using genome-wide SNP markers converted into haplotype block alleles for perpetual flowering. X-axis represents subgenomes. **(a)** posterior probability plot, and **(b)** traces of QTL models.


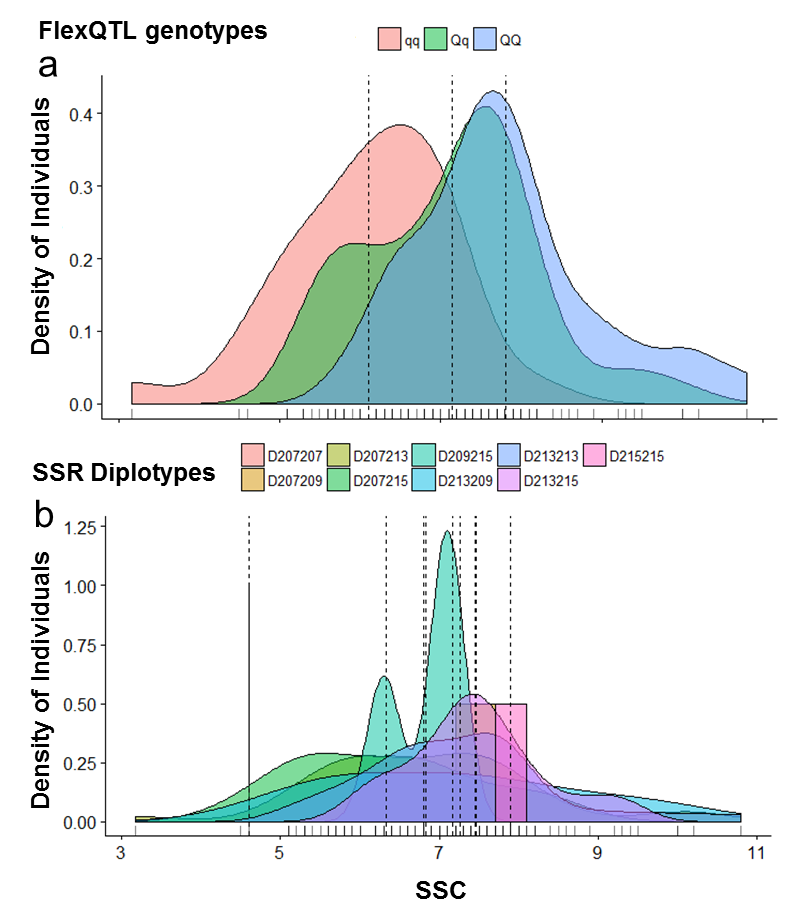


**Supplementary Figure S9.** Distributions of soluble solids content associated with FlexQTL assigned QTL genotypes and SSR marker EMFv006 based genotypes for OR-2012. **a**) density plots of SSC associated with FlexQTL assigned QTL genotypes at the SSC QTL locus on LG 6A. Dotted vertical lines represent mean of the distribution and colors represent different FlexQTL assigned QTL genotypes (*qq*, *Qq*, *QQ*). **b**) density plots of SSC associated with SSR marker EMFv006 genotypes. Dotted vertical lines represent mean of the distribution and colors represent different SSR marker genotypes.


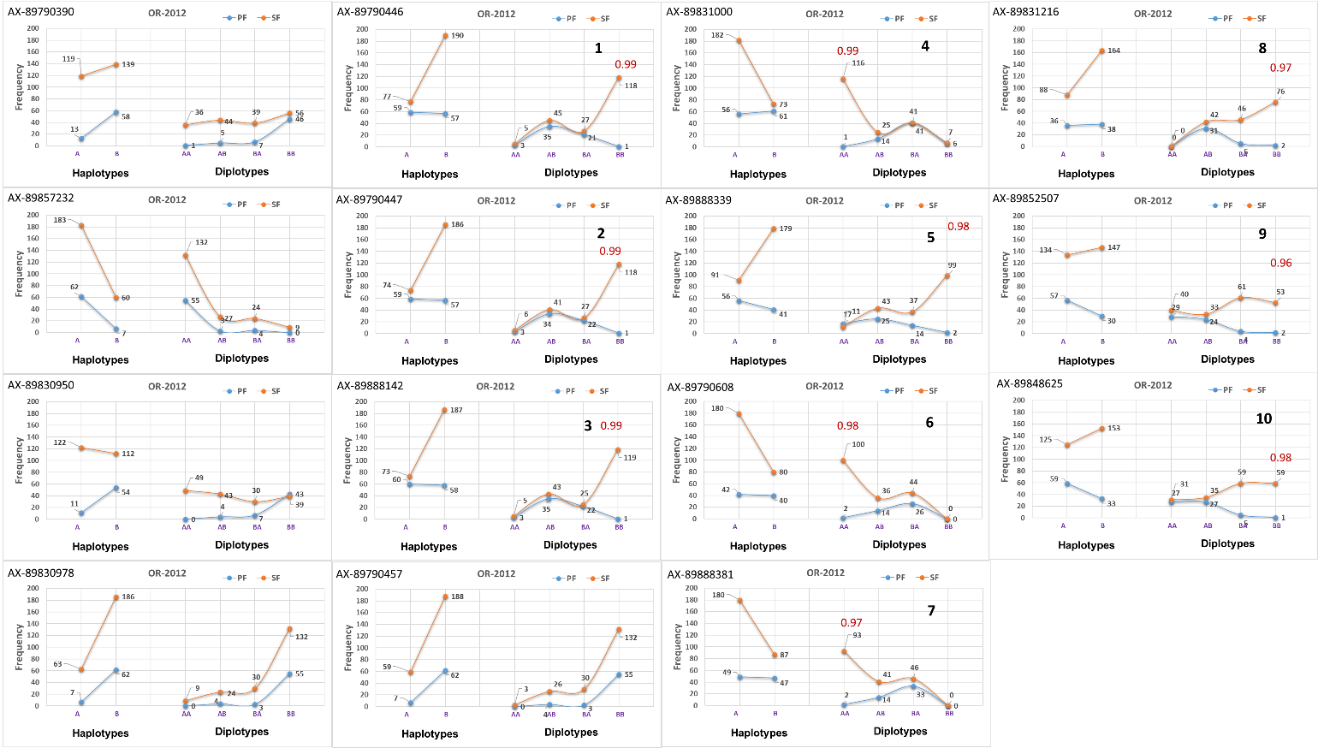


**Supplementary Figure S10.** Association of haplotypes and diplotypes of each of the 15 SNP markers with flowering in the OR 2012 dataset. Patterns were similar for other years and locations. Numbers 1-10 identify SNP markers with higher predictive ability for seasonal flowering (SF) compared to perpetual flowering (PF)
